# Evaluation of mobile phone‐based Positive Deviance/Hearth child undernutrition program in Cambodia

**DOI:** 10.1111/mcn.13224

**Published:** 2021-08-19

**Authors:** Melissa F. Young, Diane Baik, Kathryn Reinsma, Lucas Gosdin, Hannah Paige Rogers, Sreymom Oy, Wuddhika Invong, Heang Hen, Sopheap Ouk, Chhea Chhorvann

**Affiliations:** ^1^ The Hubert Department of Global Health, Rollins School of Public Health Emory University Atlanta Georgia USA; ^2^ Technical Service Organization World Vision International Monrovia California United States; ^3^ National Institute of Public Health Phnom Penh Cambodia; ^4^ World Vision International—Cambodia Phnom Penh Cambodia

**Keywords:** Cambodia, children, Positive Deviance/Hearth programme, underweight

## Abstract

Child undernutrition in Cambodia is a persistent public health problem requiring low‐cost and scalable solutions. Rising cellphone use in low‐resource settings represents an opportunity to replace in‐person counselling visits with phone calls; however, questions remain on relative effectiveness. Our objective was to evaluate the impact of two options for delivering a World Vision infant and young child feeding (IYCF) counselling programme: (1) traditional Positive Deviance/Hearth (PDH) programme with in‐person visits or (2) PDH with Interactive Voice Calling (PDH‐IVC) which integrates phone calls to replace 62.5% of face‐to‐face interaction between caregivers and volunteers, compared to the standard of care (SOC). We conducted a longitudinal cluster‐randomised controlled trial in 361 children 6–23 months. We used an adjusted difference‐in‐difference approach using baseline, midline (3 months) and endline (12 months) surveys to evaluate the impact on child growth among the three groups. At baseline, nearly a third of children were underweight, and over half were food insecure. At midline the PDH group and the PDH‐IVC groups had improved weight‐for‐age z‐scores (0.13 DID, p = 0.011; 0.13 DID, p = 0.02, respectively) and weight‐for‐height z‐score (0.16 DID, p = 0.038; 0.24 DID, p = 0.002), relative to SOC. There were no differences in child height‐for‐age z‐scores. At endline, the impact was sustained only in the PDH‐IVC group for weight‐for‐age z‐score (0.14 DID, p = 0.049), and the prevalence of underweight declined by 12.8 percentage points (p = 0.036), relative to SOC. Integration of phone‐based IYCF counselling is a potentially promising solution to reduce the burden of in‐person visits; however, the modest improvements suggest the need to combine it with other strategies to improve child nutrition.

**Key messages**

- The traditional Positive Deviance/Hearth programme (PDH) and Positive Deviance/Hearth with Interactive Voice Calling (PDH‐IVC) (replace in‐person follow‐up visits with mobile phone calls) infant and young child feeding behaviour change programmes had a modest positive effect on child nutrition after 3 months with some evidence of sustained impact at 12 months in the PDH‐IVC group.
- Integration of mobile phone‐based counselling is a potentially promising solution to reduce the burden and potential person‐to‐person exposure of in‐person visits for programmes.
- Despite modest improvements during the programme, the prevalence of underweight among young children remains unacceptably high and requires further investment.

## 1 INTRODUCTION

Child undernutrition is the leading cause of poor health outcomes and child death among children under 5 years within low‐ and middle‐income countries (WHO, 2018). To meet Sustainable Development Goals (UNICEF, 2015a) for reducing child mortality and improving child nutrition status, concerted efforts must be made to address the underlying determinants of undernutrition, which include household food insecurity, inadequate care and feeding practices and unhealthy household environments (UNICEF, 2015b). Bhutta et al. estimates that 1 million lives could be saved if access to evidence‐based nutrition interventions were scaled up to 90% coverage (Bhutta et al., 2013). Developing innovative delivery strategies can reduce the human and financial resources that are often barriers to scaling nutrition interventions. This is especially pertinent in light of the COVID‐19 pandemic in which people are infected at high rates through person‐to‐person exposure.

COVID‐19 is expected to exacerbate child undernutrition due to considerable declines in household incomes and reduced accessibility and affordability of nutritious foods (D. Headey et al., 2020). According to Headey et al., the number of people in lower‐ and middle‐income countries facing acute food insecurity is expected to double to 265 million by the end of 2020 (D. Headey et al., 2020). Unfortunately, many existing interventions have stopped altogether due to the evacuation of service providers or ethical responsibility to avoid the continued spread of COVID‐19. Service providers are thus challenged with developing low‐ or no‐contact solutions to provide ethical, health and nutrition services to women and children, especially in low‐income and low‐resource settings. There is a need for more evidence on how service providers can minimise face‐to‐face interaction of caregivers and health workers while maintaining the effectiveness of child nutrition programmes.

Despite rapid economic growth, increased donor funding and improvement in quality of maternal and child health care services, according to the 2014 Cambodia Demographic and Health Survey (CDHS) the prevalence of stunting (height‐for‐age z‐score < −2.0), wasting (weight‐for‐height z‐score < −2.0) and underweight (weight‐for‐age z‐score< −2.0) among children under age 5 has remained unacceptably high at 32%, 10% and 24%, respectively (Mallick et al., 2018). The Royal Government of Cambodia, with the support of UNICEF and the World Food Program, estimates that undernutrition has caused an annual loss of over USD 250–400 million to Cambodia's gross domestic product (CARD, UNICEF. World Food, 2013). In an effort to improve the health of Cambodian children, World Vision International‐ Cambodia (WVI‐C) has employed evidence‐based nutrition programming under the ‘Positive Deviance/Hearth (PDH)’ approach.

PDH is a community‐based intervention that identifies major challenges contributing to undernutrition in the community and finds local solutions to address them by observing the positive behaviours practised in ‘positive deviants’, children from poor‐resource households with better nutritional outcomes compared to peers in the community. The positive behaviours identified are locally appropriate IYCF; water, sanitation and hygiene (WASH); child‐caring; and health‐seeking practices that are designed into six contextualised messages shared through a volunteer‐led 10–12 day education session where caregivers with underweight children practice cooking and feeding their children micronutrient dense meals, promoting positive behaviour change and rehabilitation of the underweight child (Marsh et al., 2004). Participation in the PDH has been associated with improved nutrition and feeding knowledge of mothers and reductions in severe cases of underweight among young children (Marsh et al., 2004). However, as highlighted in a PDH systematic review there are challenges in interpreting existing evidence given the use of pre‐post designs without a control group, non‐randomised design and small sample sizes (Bisits Bullen, 2011). In addition, programme results may be context‐specific and depend on programme intensity and the level of implementation (Bisits Bullen, 2011).

Programmatic costs and volunteer burden have also been identified as a barrier to scalability. To increase scalability and reduce volunteer burden in PDH programming, we modified the PDH package by integrating PDH with mobile phone technology. Although there is some promising research to suggest short‐message service/text reminders and voice recordings may have a positive impact on health behaviour change (Yasmin et al., 2016), no prior research has investigated the effectiveness of replacing in‐person home visits with direct phone calls from PDH volunteers within PDH programmes. This study aimed to evaluate the impact of two options for delivering a World Vision infant and young child feeding (IYCF) counselling programme: 1) traditional Positive Deviance/Hearth (PDH) programme with in‐person visits or 2) Positive Deviance/Hearth with Interactive Voice Calling (PDH‐IVC) programme which integrates phone calls to replace 62.5% of face‐to‐face interaction between caregivers and volunteers, on reducing the prevalence of underweight compared to the control group receiving the standard of care (SOC: the standard government Basic Health and Nutrition Package known as ‘5 + 5 + 5’).

## 2 MATERIALS AND METHODS

### 2.1 Study overview

A longitudinal cluster‐randomised controlled trial was conducted in three districts located in the Kampong Chhnang and Kampong Speu Provinces of Cambodia. The project was led by World Vision with technical support from Emory University and the National Institute of Public Health (NIPH). We purposively selected three area programmes (APs) with similar socio‐demographic and health characteristics; AP and PDH start dates; underweight prevalence rates; food security; peri‐urban setting; migration rates; and community context (women primarily working in garment factories, households primarily rice farmers, comparable living standards and access to health care). From within the APs, 24 health centre coverage areas (clusters) selected by probability proportional to the population of children 6–24 months of age were randomised into one of the three intervention groups. Children were randomly selected after screening for a weight‐for‐age z‐score < −1.

All three intervention groups received Cambodia's Basic Health and Nutrition Package (5 + 5 + 5) for pregnant and lactating women and children under 2 years of age as described in Supporting Information Table S1. The *standard of care (SOC) group* received only the 5 + 5 + 5 package implemented through the Village Health Support Group/Community Health Workers and/or Traditional Birth Attendants with the support of Provincial Health Department Officers, Operational Health District officers and Health Centre Staff. Training is provided by the Ministry of Health for interventions including antenatal care, postnatal care and community‐integrated management of childhood illnesses. 5 + 5 + 5 provides basic maternal and child health support in three primary areas: service and supplies for mothers, service and supplies for children and household behaviours.

The PDH group received 5 + 5 + 5 plus the in‐person PDH intervention. In PDH, formative research is conducted through a situational analysis and Positive Deviance Inquiry (PDI) that seeks to identify the major challenges in the community contributing to undernutrition and the positive health, hygiene, caring and feeding practices in local low‐resource families whose children are well‐nourished despite living in the same context and food environment as other families. During the PDI, the feeding and childcare practices of the positive deviant children were documented. Areas of interest included local foods that improve the nutrient density of meals and proactive, culturally appropriate child feeding practices, such as active feeding and food hygiene practices. From the PDI findings, six contextualised key messages addressing the major challenges contributing to undernutrition in the community were streamlined and shared during the Hearth sessions. Examples of these messages included feeding children a balanced diet – especially focusing on examples of locally available low‐cost protein‐rich foods and Vitamin A‐ and C‐rich vegetables to be added to children's meals; types of micronutrient‐rich vegetables to grow at home; frequent feeding of children with healthy snack options; keeping the home environment clean; handwashing and bathing practices; food preservation techniques; how to feed children when sick; and responsive feeding. The positive practices were taught to caregivers of underweight children in an intensive, volunteer‐led 10‐day behaviour change programme known as ‘Hearth’ sessions. The in‐person Hearth sessions would last 2 to 3 h for consecutive days. The meal given during the Hearth session was an ‘extra’ meal and not a replacement meal, assisting in the nutritional rehabilitation of the children. The Hearth meal varied for each session and menus were designed using locally available and accessible foods and developed based on the PDI findings. During each daily session, the caregivers learned one or two of the six key Hearth messages as they practised cooking the menus, cleaning, caring and feeding the children together. Additional health and nutrition messages from the 5 + 5 + 5 core package were also shared. Throughout the implementation of PDH, emphasis was placed on providing targeted feeding and messages, providing caregivers an opportunity to model and practice the behaviours promoted during the session. Following the Hearth sessions, there were 2 weeks of in‐person home follow‐up visits (2–3 days/week) to help reinforce key messages and follow up with caregivers.

The PDH‐IVC group was identical to the PDH programme described above with two exceptions (Supporting Information Table S2). The 10 days of Hearth sessions were replaced by 5 days of in‐person sessions during Week 1 and 5 days of educational phone calls by the volunteers during Week 2. The Hearth meals were encouraged to be cooked and fed to the Hearth participant children at home during the 5 days of phone calls by the volunteers, which lasted an average of 15 min per call. The 2–3 days of home follow‐up visits were replaced by 5 days of follow‐up phone calls in Week 3, two more days of voice calls in Week 4 by the volunteers and a final home follow‐up visit to weigh the child at the end of Week 4. The home follow‐up visits and voice calls were used to follow up with PDH participant caregivers to (i) help overcome barriers faced at home in practising the positive behaviours learned during the PDH sessions, (ii) reinforce the new behaviours at home, (iii) provide reminders to practice the new behaviours at home and (iv) help identify the positive changes seen in their children, which helps reinforce the importance of continuing the changed positive behaviours at home. At the end of the 4‐week programme for both PDH and PDH‐IVC the child was weighed and if they have not gained sufficient weight, they were invited to repeat the programme for another round.

### 2.2 Data collection

Baseline, midline (3 months) and endline (12 months) data collection was completed by the NIPH with guidance from Emory University and World Vision International Technical Services Organization (WVI TSO), with in‐country support provided by World Vision International – Cambodia (WVI‐C). Mobile tablets and phones were used for data collection on the Open Data Kit platform. All tools were translated to Khmer from English.

The software programme PASS, Power Analysis and Sample Size Software (NCSS, LLC. Kaysville, Utah) was used to calculate sample size for cluster‐randomised trials. Within the statistical parameters of 80% power (β = 0.20), a 95% confidence interval (α = 0.05) and a difference of at least 15 percentage points for the prevalence of underweight, sample sizes of 90 per each of the three groups was determined. Accounting for 35% attrition (a conservative attrition rate was utilised to account for the high migration rate for the context), 120 children with WAZ < −1.0 per group was sampled for a total sample size of 360.

Baseline data collection was conducted over 2 weeks during November/December 2017. The midline data collection was conducted in six waves based on the timing of implementation. The first wave of PDH intervention began 12 December 2017; therefore, the first wave of midline data collection was on 11 March 2018 (all midline data collection was conducted on the 90th day from Day 1 of when the child was enrolled in the hearth session). Endline data collection was conducted at 12 months during January and February 2019.

### 2.3 Household questionnaire

The household questionnaire was translated into Khmer and pre‐tested to ensure the appropriateness of questions prior to their use. The questionnaire included socioeconomic and demographic information; caregivers' knowledge, attitudes, practices, household food security, children's health and programme participation. To assess the nutritional status of the children, anthropometric measurements of weight, height and mid‐upper arm circumference (MUAC) were collected during household visits using standard procedures. Weight was measured in kilograms using the UNICEF Uniscale with precision to 100 g. Length was measured in centimetres using a length board (UNICEF/WFP) with precision to 1 millimetre. MUAC tape was used to measure the child's mid‐upper arm circumference in centimetres, with precision to 1 millimetre. All the enumerators were trained and performed anthropometric measurements during the survey pilot to assess their skills prior to data collection at baseline, midline and endline. Weight measurement was read and recorded twice by the measurer and assistant. Height/length measurement was taken by the measurer two separate times. If the first and second measurements had a difference greater than 0.5 cm, 1.0 cm and 0.3 kg for MUAC, length/height and weight, respectively, a third measurement was performed.

### 2.4 Data analysis

The WHO Anthro SAS Macro was used to calculate weight‐for‐age (WAZ), weight‐for‐height/length (WLZ) and height/length‐for‐age (LAZ) z‐scores based on the WHO Child Growth Standards (WHO, 2006). Standard cut‐offs for stunting, wasting and underweight were used (< −2 LAZ, < −2 WLZ and < −2 WAZ, respectively). Outliers for WAZ, WLZ and LAZ were excluded according to the WHO flags for biologically implausible values (Freedman et al., 2016). At endline, one implausible value of WLZ at baseline was removed (< −5 SD). An additional 4 values of height at baseline and one at midline were removed because they were not consistent with other measurements for the individual (i.e., height declined with time). In addition, Emergency Nutrition Assessment (ENA) Software was utilised to conduct data plausibility checks to ensure biological credibility of anthropometric measurements, no duplication of records and confirm the quality of data. Wealth tertiles were calculated following principal components analysis of housing materials, water and sanitation facilities and household income (Vyas & Kumaranayake, 2006). Standard IYCF indicators were used including timely introduction of complementary foods, minimum dietary diversity and minimum meal frequency (World Health Organization, 2008).

All quantitative data analyses were performed using SAS version 9.4 (SAS Institute, Cary, NC). Complex survey procedures were used for all analyses to account for clustering within the health centre coverage area. Descriptive data from surveys on household characteristics and child feeding practices were summarised using means and frequencies and examined differences among groups using ANOVA with Taylor series variances and Rao–Scott chi‐square tests. Linear mixed‐effects models were used to model the changes in the intervention group from baseline to endline relative to the control group, also called the difference‐in‐difference (DID). Despite randomisation at the cluster level, we noted differences within each treatment group in the outcomes of interest at baseline. A comparison of differences in the outcomes at each follow‐up would have ignored the changes from baseline values. The DID analytical approach accounts for differences at baseline and changes over time [(intervention endline – control endline) ‐ (intervention baseline – control baseline)]. Crude and adjusted models were used to compare the change in each intervention group from baseline to midline and baseline to endline relative to the change in the control group. In adjusted models, potential confounding variables were included as fixed effects because they differed between groups at baseline. These covariates included caregiver education, household wealth and household food security. With this analytical approach we assume that, though baseline differences exist, trends in the outcomes would be similar in the absence of intervention. Inclusion of potential confounders in the adjusted models and the small geographical area of the study help to support this assumption. A p‐value less than 0.05 was considered significant.

### 2.5 Ethical considerations

Ethical approvals were sought from the Cambodian National Ethic Committee and University of Emory Ethics Board. Oral informed consent was sought prior to starting the research. Participation in research was optional and did not impact the families' access or participation to community health and nutrition services. The study was registered at clinicaltrials.gov as NCT03399058.

## 3 RESULTS

**FIGURE 1:**
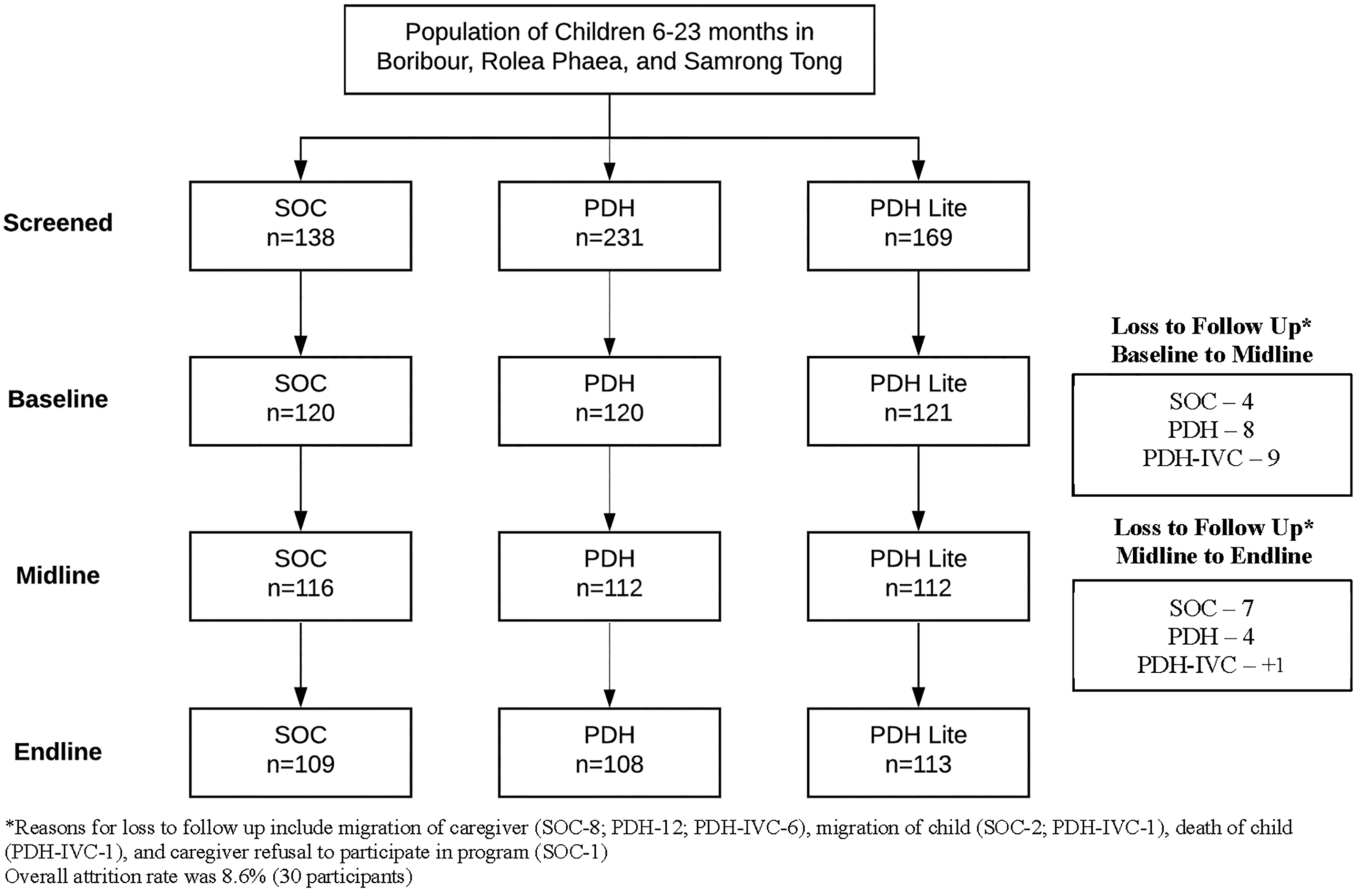
Summary participant flow chart

The total sample from baseline, midline (3 months) and endline (12 months) was 361, 340 and 330, respectively (Figure 1). The overall attrition rate was 8.6%. Reasons for loss to follow‐up included migration due to family reasons or seasonal work, hospitalisation, accident, or caregivers refusing to participate. Basic demographic characteristics of children across the three groups are described in Table 1. Most children participated in two rounds of PDH or PDH‐IVC (Table 2). Children in the PDH group attended more in‐person Hearth sessions (15.3 vs. 9.4 in PDH‐IVC), whereas children in the PDH‐IVC group had nearly twice the number of follow‐up calls (21.1 ± 1.1) compared to the number of follow‐up home visits in the PDH group (11.5 ± 0.5) throughout the total intervention period. Attendance across the three potential rounds of PDH and PDH‐IVC was similar with over 50% of participants meeting minimum attendance adherence criteria. Overall participation adherence tended to be higher in the PDH‐IVC group, though differences were not statistically different. Table 3 summarises changes in child feeding practices across the three groups during baseline, midline and endline. At baseline, most children received the minimum recommended meal frequency and had appropriate handwashing practices (> 85%). However, dietary diversity was suboptimal in all groups, with only 49.1%, 40.3% and 55.9% of children in the SOC, PDH and PDH‐IVC groups, respectively, meeting the minimum dietary diversity recommendations. In the PDH group at midline minimum dietary diversity improved by 16.3 percentage points and minimum acceptable diet improved by 23 percentage points, relative to the standard of care. There were no significant differences in feeding practices in the PDH‐IVC group. At endline there were no sustained changes in IYCF practices across the groups.

**TABLE 1:** Basic characteristics of caregivers and children at baseline (n = 361)

Characteristics	SOC	PDH	PDH‐IVC	p value*
Child's age (months)	15.9 (0.3)	15.2 (0.2)	14.9 (0.4)	0.05
Child's sex				0.30
Male	54.2	50.0	57.0	
Female	45.8	50.0	43.0	
Primary caregiver				0.01
Mother	72.5	71.7	50.4	
Father	4.2	8.3	6.6	
Grandmother	21.7	18.3	36.4	
Other relative	1.6	1.7	6.6	
Age of primary caregiver (years)	35.4 (1.3)	35.1 (1.1)	39.9 (2.4)	0.24
Education of primary caregiver (%)				0.01
Never attended	13.3	7.5	20.3	
Pre‐school or primary school	42.5	49.2	44.1	
Secondary school	31.7	38.3	31.4	
Post‐secondary	12.5	5.0	4.2	
Number of people living in the household	5.3 (0.2)	6.2 (1.0)	5.5 (0.2)	0.67
Source of income (%)				0.27
Agriculture/husbandry	7.5	5.8	7.4	
Service/salaries	24.2	39.2	45.5	
Business/trading	16.7	20.8	11.6	
Labour	39.2	25.8	24.0	
Other	12.5	8.3	11.6	
HFIAS (%)				0.02
Food secure	26.7	40.0	44.6	
Mildly food insecure	14.2	20.8	15.7	
Moderately food insecure	36.7	20.0	16.5	
Severely food insecure	22.5	19.2	23.1	
Income quintile (monthly income, USD)				0.49
1 (5–118)	12.5	20.8	22.3	
2 (123–160)	26.7	21.7	15.7	
3 (162–235)	20.8	20.0	15.7	
4 (245–217)	20.0	18.3	24.8	
5 (442–589)	20.0	19.2	21.5	

*Note:* Values are mean (SE) or %.

Abbreviations: PDH, Positive Deviance/Hearth; SOC, standard of care.

* p value for difference among groups; Rao–Scott chi‐square or ANOVA with Taylor series variance.

**TABLE 2:** Programme participation in PDH and PDH‐IVC communities

Indicator	PDH (n = 120)	PDH‐IVC (n = 121)	p value*
Number of intervention rounds attended (%)			0.05
1	18.3	30.6	
2	68.3	65.3	
3	13.3	4.1	
Total number of in‐person PDH sessions over 1 year	15.3 (0–30)	9.4 (0–15)	<0.001
Number of Round 1 sessions attended among Round 1 participants	7.4 (0–10)	4.3 (0–5)	<0.001
Number of Round 2 sessions attended among Round 2 participants	7.3 (1–10)	4.3 (0–5)	<0.001
Number of Round 3 sessions attended among Round 3 participants	7.4 (2–10)	3.8 (0–5)	<0.001
Total number of home visits (PDH) or calls (PDH‐IVC) over 1 year	11.5 (0–18)	21.1 (0–36)	<0.001
Number of Round 1 home visits/calls received among Round 1 participants	5.3 (0–6)	9.6 (0–12)	<0.001
Number of Round 2 home visits/calls received among Round 2 participants	5.5 (0–6)	9.5 (0–12)	<0.001
Number of Round 3 home visits/calls received among Round 3 participants	5.7 (4–6)	8.8 (0–12)	<0.001
Minimum adherence to the intervention^a^ (%)			
Minimum adherence in Round 1 among Round 1 participants	64.2	74.4	0.27
Minimum adherence in Round 2 among Round 2 participants	51.2	67.9	0.19
Minimum adherence in Round 3 among Round 3 participants	56.7	55.4	0.90

*Note:* Values are % or mean (minimum–maximum).

Abbreviations: PDH = Positive Deviance/Hearth.

^a^ Minimum adherence was defined per round as attending a minimum of eight in‐person PDH sessions and receiving four or more home visits for the PDH group and attending a minimum of four in‐person PDH sessions and receiving eight or more phone calls for the PDH group. In PDH, 120 children participated in Round 1, 82 children participated in Round 2 and 16 children participated in Round 3. In PDH‐IVC, 121 children participated in Round 1, 79 children participated in Round 2 and 5 children participated in Round 3.

* Rao–Scott chi‐square test and *t* test with Taylor series variance estimation for differences in the distribution of values and mean, respectively, between the two intervention groups.

**TABLE 3:** Adjusted difference in difference in infant and young child feeding practices (IYCF) and child growth

Indicator	Baseline (%) (n = 361)			Midline (%) (n = 340)					Endline (%) (n = 330)				
	SOC	PDH	PDH‐IVC	SOC	PDH	PDH‐IVC	DID^a^	DID^b^	SOC	PDH	PDH‐IVC	DID^c^	DID^d^
IYCF indicators													
Minimum dietary diversity	49.1	40.3	55.9	75.7	83.3	86.1	16.3*	3.6	88.9	86.5	87.3	6.4	−8.4
Minimum meal frequency	86.5	86.3	87.9	60.5	70.9	68.5	10.6	6.5	14.8	16.8	20.8	2.2	4.5
Minimum acceptable diet	44.9	36.7	56.0	46.3	61.2	59.8	23.1*	2.4	15.5	14.5	15.1	7.2	−11.6
Appropriate handwashing	88.3	84.5	87.3	89.1	86.8	90.5	1.5	2.4	97.2	96.9	97.2	3.6	1.0
Child anthropometry													
Underweight (< −2 WAZ)	27.2	29.9	33.4	37.3	34.2	34.6	−5.8	−8.8	36.3	35.5	29.7	−3.6	−12.8*
Wasting (< −2 WHZ)	11.5	20.0	10.5	12.7	16.1	12.1	−5.1	0.4	10.7	14.8	9.3	−4.4	−0.4
Stunting (< −2 HAZ)	39.0	28.6	31.1	47.2	34.8	46.2	−2.1	6.8	47.2	35.1	41.0	−1.8	1.7
													

Abbreviations: HAZ, height/length‐for‐age z‐score; PDH, Positive Deviance/Hearth; SOC, standard of care; WAZ, weight‐for‐age z‐score; WHZ, weight‐for‐height/length z‐score.

^a^ Difference in difference (percentage points) in PDH from baseline to midline relative to the SOC, adjusted for baseline food security, caregiver education and wealth.

^b^ Difference in difference (percentage points) in PDH‐IVC from baseline to midline relative to the SOC, adjusted.

^c^ Difference in difference (percentage points) in PDH from baseline to endline relative to the SOC, adjusted.

^d^ Difference in difference (percentage points) in PDH‐IVC from baseline to endline relative to the SOC, adjusted.

* p < 0.05.

**FIGURE 2:**
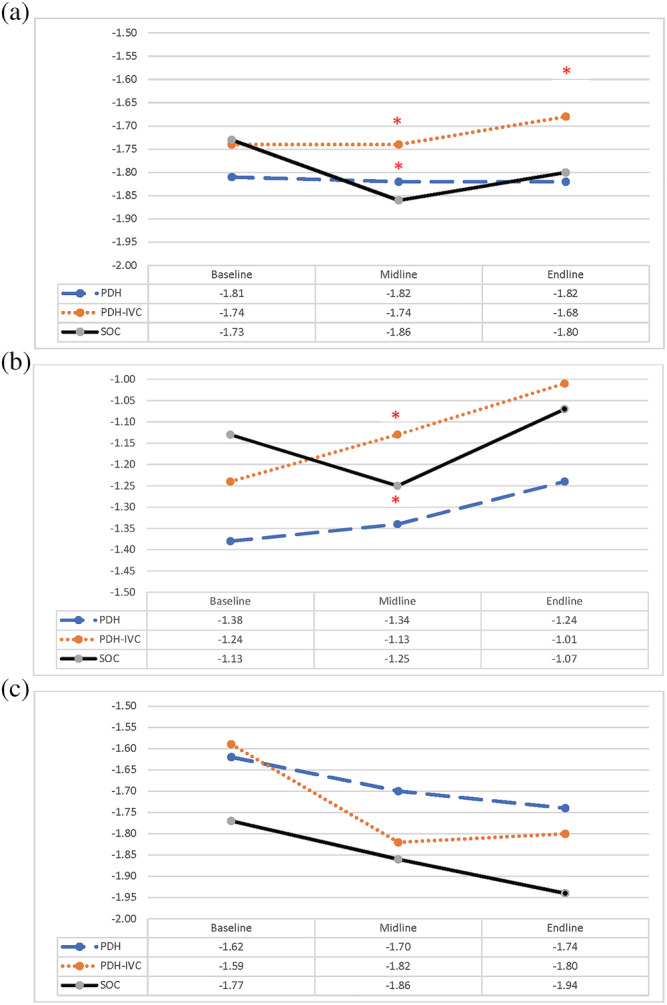
Adjusted difference in difference for (a) weight‐for‐age Z‐score, (b) weight‐for‐height/length Z‐score and (c) height/length‐for‐age Z‐score (n = 358). Models are adjusted for caregiver education, household wealth and household food security. Significance denoted by * at p < 0.05. In (a): PDH versus SOC DID midline: 0.13 (p = 0.01) and endline: 0.07 (p = 0.29); PDH‐IVC versus SOC DID midline: 0.13 (p = 0.02) and endline: 0.14 (p = 0.049). In (b): PDH versus SOC DID midline: 0.16 (p = 0.04) and endline: 0.09 (p = 0.40); PDH‐IVC versus SOC DID midline: 0.24 (p = 0.002) and endline: 0.18 (p = 0.07). In (c): PDH versus SOC DID midline: 0.00 (p = 0.96) and endline: 0.04 (p = 0.65); PDH‐IVC versus SOC DID midline: −0.14 (p = 0.18) and endline: −0.05 (p = 0.70)

Figure 2a illustrates the adjusted difference‐in‐difference (DID) results for child WAZ at all three time points. WAZ remained fairly constant from baseline to midline in the PDH (−1.81 to −1.82) and the PDH‐IVC (−1.74 to −1.74) groups; however, in the SOC group, the WAZ declined from −1.73 to −1.86. Thus, providing some evidence of a protective effect in intervention communities. The adjusted mean differences for WAZ for the PDH and PDH‐IVC groups relative to the SOC group at midline were respectively 0.13 DID (p = 0.01) and 0.13 DID (p = 0.02). At endline, differences remained significant only for the PDH‐IVC group relative to the SOC (0.14 DID, p = 0.049). There were likewise significant improvements in child WLZ at midline in the PDH (WLZ, baseline −1.38 to midline −1.34; 0.16 DID, p = 0.04) and PDH‐IVC group (WLZ baseline −1.24 to midline −1.13; 0.24 DID, p = 0.002), whereas weight‐for‐length/height z‐scores declined in SOC (WLZ baseline −1.13 to midline −1.25), Figure 2b. However, the children in the intervention groups were no different than those in the standard of care at the 1‐year follow‐up. Child LAZ declined across the project time period, with no significant differences across groups, Figure 2c. There were no significant differences in the relative change in wasting or stunting across groups; however, the prevalence of underweight was reduced by 12.8 percentage points in the PDH‐IVC group at endline, relative to the SOC (Table 3). There were also no significant differences in the relative change MUAC across groups (data not shown).

## 4 DISCUSSION

The in‐person PDH and mobile phone‐integrated PDH‐IVC programmes had a modest positive effect on child nutrition (improved WAZ and WLZ) at 3 months with limited evidence of sustained impact in the PDH‐IVC group at 1 year. Our study provides novel findings on the effectiveness of replacing volunteer and caregiver face‐to‐face interactive sessions with phone calls to reduce the work burden of volunteers. Our results are particularly timely given the current COVID‐19 crisis and the need for programmes to adapt to challenging environments and reduce in‐person exposure for both households, community volunteers and health care workers.

Our findings align with prior PDH research demonstrating a positive impact on child nutritional status, though improvements were less than anticipated. The intervention appeared to have a protective effect against the rising rates of child undernutrition noted in the SOC group during baseline to midline. The declines in child WAZ and WHZ may be due to seasonal food insecurity or age‐related rise in undernutrition. Much of the existing evidence for PDH programmes is based on observational programme monitoring data. For example, in a PDH programme led by STRIDES funded by USAID in Uganda, 4,501 underweight children 6 to 59 months were enrolled in the programme over 3 years resulting in 68% gaining 400 g or more after 26 Hearth days (STRIDES, 2015). Each year, the percentage of children enrolled who gained at least 400 g within the first 26 days increased from 59.5% to 66.2% to 72.7% (STRIDES, 2015). Observational PDH studies from Zambia and Burundi showed large reductions in the prevalence of underweight in communities where PDH had been implemented. Adequate weight gain in PDH interventions was defined as weight gain of greater than or equal to 200 g in 12 days, 400 g in 30 days and 900 g in 3 months (Sosanya et al., 2017). A PDH intervention in Zambia with a small sample size (n = 9) resulted in 89%, 100% and 100% of participants gaining adequate weight at 12, 30 and 90 days, respectively (Mulenga, 2014). The Burundi PDH intervention (n = 1941) reported that 87% gained adequate weight at 12 days with sustained weight gain among the majority of participants at 1 month (CRS Burundi, 2012). Although existing evidence on sustained PDH impact is limited, our findings are in alignment with another PDH cluster‐randomised trial in Ethiopia, which resulted in a 6.3% decrease in underweight at 1 year (Kang et al., 2017). In our study, we observed a 12.8 percentage point reduction in underweight in the PDH‐IVC group relative to the SOC at the 1‐year follow‐up (results for traditional PDH programme were non‐significant).

Key differences between the PDH‐IVC and traditional PDH groups may help to explain why we saw better outcomes in the PDH‐IVC group. Although the PDH group received more in‐person Hearth sessions than the PDH‐IVC group (15.3 vs. 9.4), the PDH‐IVC group received nearly twice the number of follow‐up sessions (21.1 ± 1.1 vs. 11.5 ± 0.5) indicating that the follow‐up phone calls may have been more effective or easier to conduct compared to the in‐person follow‐ups. The difference in biweekly in‐person follow‐up in the traditional group compared to nearly daily phone calls may have provided caregivers with more consistent and timely reminders as they engaged in nutrition behaviour changes in the first few weeks. Additionally, because the PDH‐IVC volunteers did not have to spend time travelling from home to home, there may have been more quality time dedicated to each caregiver during the mobile phone follow‐ups.

There is a need to continue evaluating the effectiveness of mobile phone‐based nutrition interventions; however, the initial promising results from this study could have important implications for the current COVID‐19 environment. According to *Sight and Life*, 97% of the world's population has access to a mobile phone signal, and while access to a mobile phone is still improving, the inevitability of mobile phone usage on a global scale has important potential for use in innovative solutions against undernutrition (Bhatt, 2017). In this peri‐urban context, cell phone use was already widespread among both families and community volunteers. The IVC component relied on tailored voice calls using simple mobile phones, not SMS or smartphones, so the digital literacy required was quite low. However, a challenge was occasional poor mobile phone service and older caregivers not always carrying the phones with them. Mobile phones were lent out for caregivers who did not have access to mobile phones or if their household had one shared phone. Electricity was not a challenge because solar chargers were provided. Because the programme started with five in‐person Hearth sessions, volunteers were able to develop a relationship with caregivers before providing messages through phone calls. Thus, volunteers did not have challenges with caregivers avoiding their calls. Volunteers mentioned that they would have to make multiple household visits if caregivers were not home, but with phone calls, they could call again during a different time of day if programme recipients did not answer the first time. Several caregivers also mentioned that they appreciated phone calls because they could attend to other chores and work while on the phone and the one‐on‐one time over the phone with volunteers was valuable to ask questions difficult to ask in group settings. This issue and the details of deciding when phone calls should be made, lengths of calls and guide for sharing the messages over the phone will be further in a forthcoming paper examining the barriers, facilitators and contextual factors that influenced the design, implementation and utilisation of this programme.

Mobile health interventions have the potential to improve nutrition behaviours, strengthen undernutrition case records, improve treatment adherence and increase the efficiency of community health workers, health care facilities and systems providing nutrition support (Bhatt, 2017). Within the COVID‐19 environment, global health experts are calling for innovative strategies to be immediately employed to help mitigate the impending undernutrition crisis as a result of the virus. *Global Nutrition Report* has proposed five key areas for action across sectors to address nutrition as part of the global COVID‐19 response ‐ two of which call for the use of mobile technology to strengthen nutrition support and programming on a global scale (Headey & Ruel, 2020). Specifically, these include providing critical community‐based nutrition services using digital delivery systems and rolling out national communication campaigns on COVID‐19 to strengthen social distancing, breastfeeding, handwashing, maintaining healthy diets and information on access to basic nutrition and health services such as national vitamin supplement interventions and child immunisations (Shekar & Okamura, 2020). We suggest that the PDH‐IVC model can be flexibly adapted to the COVID‐19 context to provide caregivers with nutrition knowledge and support, which, combined with interventions that address the availability and accessibility of food, may help to alleviate the burden of undernutrition in the most vulnerable populations.

A key strength of our study is its design. Our study builds upon prior PDH observational findings and adds to the evidence base by conducting a more rigorous 1‐year longitudinal RCT design and DID analysis to control for societal and seasonal changes over time. This design element was critical given the changes in food security and trends of increased child undernutrition in control communities over time. Our study also had high retention and less than 10% of the cohort was lost to follow‐up. However, there are also several limitations. Randomisation at the cluster‐level did not result in similar groups at baseline. This suggests that randomisation at the individual‐level may have been more appropriate, though not feasible for the community‐based intervention. We accounted for this issue by using a DID analytical approach. Neither of the intervention groups resulted in an improvement in length‐for‐age z‐score or stunting. This may be due to both intervention design and evaluation, and a longer study duration and more intensive intervention may be required to detect changes in child linear growth. Furthermore, despite a year of both government and NGO programming on child nutrition, the prevalence of undernutrition among children remained unacceptably high in communities at endline, with nearly one in three children being underweight. This may be due to multiple factors including the high prevalence of local food insecurity, seasonality, caregiver mental health, among other unmeasured factors. Approximately 65% of participant families were experiencing some level of food insecurity throughout the study period, which likely inhibited their ability to implement the knowledge gained from the intervention. The UNFAO estimates that the average Cambodian spends more than 70% of their total yearly income on food alone with nutrient‐poor rice and grain cereals contributing approximately 68% of Cambodian's daily energy supply (UNFAO, 2011). Although the SOC, Maternal, Newborn and Child Health and Nutrition package for Cambodia, provides a number of services for women and children (described in Supporting Information Table S1) to combat the effects of food insecurity and diarrheal diseases throughout the region, the implementation of these programmes remains low. For example, at the time of the Demographic and Health Survey in 2014, only 58.7% and 69.6% of children under 5 years in Cambodia had received deworming and Vitamin A supplementation, respectively (CDHS, 2015). Additionally, although 95.8% of mothers reported knowing about oral rehydration salts (ORS) for the treatment of diarrhoea, only 55.5% sought treatment for their child during a diarrheal episode, of which 35.2% of cases were treated with ORS, and only 5% of cases were given zinc supplements, although the provision of each of these services are outlined in the SOC (CDHS, 2015).

The PDH and PDH‐IVC programmes are behaviour change interventions focused on improving child nutrition using locally appropriate IYCF counselling and feeding recommendations based on locally available nutritious and affordable foods. Despite intentional efforts to select affordable local foods for recipes and identify positive deviant behaviours that incorporated healthy coping mechanisms within this context; given the level of food insecurity, and the lack of uptake or accessibility to nutrition‐supporting services, the behaviour change programmes may not be appropriate on their own. There is a need for further research and potential pairing of the PDH programmes with a nutrition‐sensitive intervention such as, agricultural programming, microfinance education, or income‐generating activities, that work to resolve the root causes of undernutrition thereby improving economic mobility and access to dietary diversity to enhance programme effectiveness (STRIDES, 2015).

## 5 CONCLUSION

In summary, the PDH programmes had a modest positive effect on child nutrition with the greatest effects on weight‐for‐age z‐score and underweight with the PDH‐IVC group. We provide novel insight that that PDH‐IVC, which uses a combination of in‐person and phone calls for education sessions and home visits, improves child nutritional status compared to standard of care and provides a promising programme modification to reduce costs and in‐person exposure for future programmes.

## CONFLICTS OF INTEREST

The authors declare that they have no conflicts of interest.

## CONTRIBUTIONS

MFY, DB, KR, SO, HH, SO, CC: conceptualised the study design; SO, HH, SO, CC: data collection; MFY, DB, KR, LG: data analyses/interpretation; MFY, DB, KR, LG, HPR, assisted in writing and editing of the manuscript; MFY, DB, KR, LG, HPR, SO, HH, SO, CC: read and approved the manuscript.

## Supporting information

**Data S1** Supporting information

## Data Availability

The data that support the findings of this study are openly available in figshare at https://doi.org/10.6084/m9.figshare.14381888.v3
