# Supplementary material for: Evaluation of mobile phone‐based Positive Deviance/Hearth child undernutrition program in Cambodia
Source: Matern Child Nutr. 2021 Aug 19;17(4):e13224. doi: 10.1111/mcn.13224 (PMC8476410; doi:10.1111/mcn.13224)
Supplement: Supplementary file 1 — Data S1 Supporting information [file MCN-17-e13224-s001.docx]

**Supplemental Table 1: Components of the Maternal, Newborn, and Child Health and Nutrition package for Cambodia called, “5+5+5”**


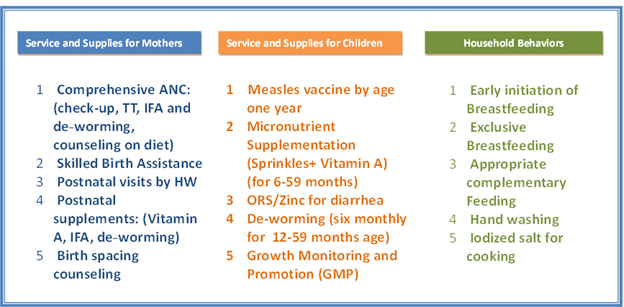


*All children in the three groups: PDH, PDH-IVC and SOC received the 5+5+5 program.

**Supplemental Table 2: Summary of PDH and PDH-IVC World Vision Child Nutrition Programs.**

|  | **PDH Program** | **PDH-IVC Program** |
| --- | --- | --- |
| **Week 1** | 5 days of in-person Hearth sessions | 5 days of in-person Hearth sessions |
| **Week 2** | 5 days of in-person Hearth session | 5 days of IYCF counseling phone calls by volunteers |
| **Week 3** | 2-3 days of in-person home follow-up visits by volunteers | 5 days of follow-up phone calls by volunteers |
| **Week 4** | 2-3 days of in-person home follow-up visits by volunteers | 2 days of follow-up phone calls by volunteers + 1 home follow-up for final anthropometric measurement |
